# Somatic, Genetic and Epigenetic Changes in Nephrogenic Rests and Their Role in the Transformation to Wilms Tumors, a Systematic Review

**DOI:** 10.3390/cancers15051363

**Published:** 2023-02-21

**Authors:** Tessa Bánki, Jarno Drost, Marry M. van den Heuvel-Eibrink, Annelies M. C. Mavinkurve-Groothuis, Ronald R. de Krijger

**Affiliations:** 1 Princess Máxima Center for Pediatric Oncology, 3584 CS Utrecht, The Netherlands; 2 Oncode Institute, 3584 CS Utrecht, The Netherlands; 3 Department of Pathology, University Medical Center Utrecht, 3584 CX Utrecht, The Netherlands

**Keywords:** nephrogenic rests, nephroblastomatosis, Wilms tumors, genetic changes, epigenetic alterations

## Abstract

**Simple Summary:**

We reviewed all studies investigating molecular changes in nephrogenic rests (NR), the presumed precursor lesions of Wilms tumors (WT) being the most frequent malignant childhood renal tumors, between 1990 and 2022. Only 23 studies were found, reporting 119 pairs of NR and corresponding WT, which may allow the detection of early genetic changes that play a role in tumorigenesis. Two genes, *WT1* and *WTX*, and two chromosomal regions, 11p13 where *WT1* is located, and 11p15 harboring the *IGF-2* gene, were found to be mutated or show loss of imprinting, respectively, in both nephrogenic rests and WT, suggesting that these could be relevant early genetic events.

**Abstract:**

Objective: To review somatic genetic changes in nephrogenic rests (NR), which are considered to be precursor lesions of Wilms tumors (WT). Methods: This systematic review is written according to the PRISMA statement. PubMed and EMBASE were systematically searched for articles in the English language studying somatic genetic changes in NR between 1990 and 2022. Results: Twenty-three studies were included in this review, describing 221 NR of which 119 were pairs of NR and WT. Single gene studies showed mutations in *WT1* and *WTX*, but not *CTNNB1* to occur in both NR and WT. Studies investigating chromosomal changes showed loss of heterozygosity of 11p13 and 11p15 to occur in both NR and WT, but loss of 7p and 16q occurred in WT only. Methylome-based studies found differential methylation patterns between NR, WT, and normal kidney (NK). Conclusions: Over a 30-year time frame, few studies have addressed genetic changes in NR, likely hampered by technical and practical limitations. A limited number of genes and chromosomal regions have been implicated in the early pathogenesis of WT, exemplified by their occurrence in NR, including *WT1*, *WTX*, and genes located at 11p15. Further studies of NR and corresponding WT are urgently needed.

## 1. Introduction

**Figure 1:**
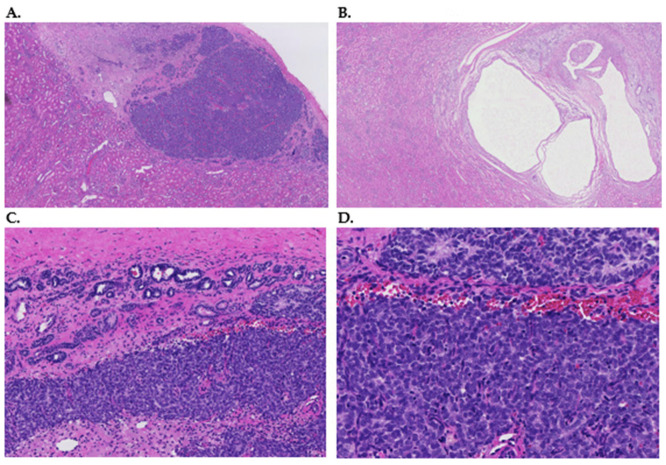
(**A**) Hematoxylin and eosin-stained slide with an overview of a hyperplastic perilobar nephrogenic rest, composed of epithelial structures without cytonuclear atypia of the composing cells. Magnification 2×. (**B**) Hematoxylin and eosin-stained slide with overview of an intralobar nephrogenic rest, composed of cystic structures with some intervening stroma. Both types of rests are surrounded by normal renal parenchyma. Magnification 2×. (**C**) Hematoxylin and eosin-stained section of a 9 mm lesion of the left kidney of 2-year-old male patient for which nephron-sparing surgery was performed. The patient underwent pre-operative chemotherapy according to the UMBRELLA protocol and also had a lesion of the contralateral kidney that was simultaneously removed. The image shows fibrous tissue of the kidney capsule at the top. Below there are glandular structures compatible with epithelial elements of a perilobar nephrogenic rest, as well as a blastemal component with limited nuclear atypia. Magnification 10×. (**D**) Hematoxylin and eosin-stained section of the same lesion as (**C**). This lesion was eventually classified as compatible with a perilobar nephrogenic rest after the careful review of multiple expert pathologists. Please note the bland nuclear morphology of the epithelium and blastema in this close-up image. Magnification 20×.

Nephrogenic rests (NR) are foci of aberrant embryonal tissue in the kidney, which are still present after 36 weeks of gestation [1,2]. They represent non-obligate precursor lesions of WT and may regress over time or progress to WT due to as yet unknown molecular factors [1,2]. The presence of multifocal or diffuse NR is referred to as nephroblastomatosis [3,4]. NR have been described as either perilobar NR (PLNR) or intralobular NR (ILNR), based on the topographic site in the kidney. Both types can also occur in the same kidney as well as in both kidneys of the same individual. PLNR are located at the periphery of the renal cortex and have been shown to consist mainly of blastemal or immature epithelial components (Figure 1A) [1,5]. ILNR are located within the lobes and are mostly composed of a combination of cystic epithelial elements surrounded by moderately cellular stromal elements (Figure 1B) [1,5,6]. In addition, heterologous components, including fat, may be present. ILNR are considered to arise prior to PLNR, and are usually not as well demarcated as PLNR [1,5,6]. Histologically, according to Beckwith’s criteria [3], NR can be subdivided into different histological subtypes such as dormant rests (especially blastemal cells with minimal proliferation), sclerosing rests (minimal blastemal components and presence of stromal maturation), hyperplastic rests (proliferative signs manifested by blastemal and epithelial cells and increased size), neoplastic rests (usually a discrete spherical nodule), and obsolete rests ((sclerotic) stromal and epithelial components, and minimal blastemal). Any histological subtype of NR has been shown to be able to transition to another NR subtype [1,4]. 

In infants, microscopic NR are identified in about 1% of autopsies, the majority of which will either remain stable or go into regression [3,7]. The occurrence of NR is partly determined by ethnic and demographic factors [6]. In White American children, 20% reveal WT-associated PLNR compared to 7% in Asian American children, and 2% in Japanese children. ILNR, on the other hand, are more common in Asian American (33%) and Japanese children (25%) relative to the White American children (17%) [8]. As ILNR seem to arise from abnormalities earlier in development, they also occur at a younger age in children, namely at a median age of 23 months, whereas PLNR are usually not discovered until a median age of 36 months [5]. PLNR occur mainly in females and ILNR are more often seen in males [6]. The age at diagnosis also differs between ethnic groups. NR are generally diagnosed earlier in Asian American patients (median age at diagnosis 31 months) than in White American patients (median age at diagnosis 39 months) [8]. 

NR have been described to lead to the development of WT in a subset of cases [3,5]. WT are the most commonly found malignant renal tumor in children [9]. WT are morphologically heterogeneous embryonic tumors, including epithelial, blastemal, stromal, and sometimes rhabdomyomatous elements [10]. Tumors can be classified in different histological types, which currently grossly determines risk stratification after neoadjuvant chemotherapy treatment [11]. The distinction between NR, mainly PLNR, and WT can be extremely challenging, both radiologically and pathologically, especially if a biopsy is submitted for evaluation. In fact, no single radiological or histological criterion, including size and shape of the lesion, can distinguish NR from WT and a 25% misdiagnosis rate has been reported for radiological assessment (see Figure 1C,D) [12,13]. PLNR are associated with epithelial, blastemal, or mixed type WT and stromal or heterologous elements are limited or absent. In contrast, there is an association with ILNR and stromal type WT and heterologous components are frequent [3]. Most WT occur sporadically and are unilateral, but they can also be familial and bilateral [6]. According to the International Society of Paediatric Oncology Renal Tumour Study Group (SIOP-RTSG), kidneys with unilateral WT contain NR in 40% of the cases (25% PLNR, 9% ILNR, 5% both PLNR and ILNR and 1% nephroblastomatosis). Bilateral WT are significantly more frequently associated with NR, as NR have been reported in 94% of the stage V cases [14].

Bilateral WT occur significantly more often in syndromes in the context of which WT recurrently occur [11]. It is beyond the scope of this review to discuss these syndromes, except for their association with NR. Examples are Denys–Drash and WAGR syndrome, which are associated with the presence of ILNR. Patients with these syndromes have a risk of about 30% and 95%, respectively, to develop WT [6]. Patients with these syndromes have a risk of up to 95% and 50%, respectively, to develop WT [15,16]. PLNR occur more frequently in patients with overgrowth syndromes such as Beckwith–Wiedemann syndrome (BWS) [6]. Some of the abovementioned syndromes have a known genetic driver, such as the occurrence of germline *WT1* mutations in Denys–Drash and WAGR syndrome [11,17]. In addition, numerous recurrent somatic genetic abnormalities, both gene mutations and copy number variations (CNV) at specific chromosomal locations, as well as hypermethylated regions have been found in non-syndromic, sporadic WTs. These include mutations in *WT1*, *CTNNB1*, *MYCN*, *TP53*, *AMER1*, *FBXW7*, *GPC3*, *MLLT1*, *DIS3L2*, *DICER1*, *DROSHA*, *DGCR8*, *SIX1* and *SIX2*, *SMARCA4*, *ARID1A* and chromosomal aberrations such as gain of chromosome arm 1q, and loss of 16q and 1p [11,18].

Although knowledge on tumor-driving changes in WT is increasing, the genetic changes underpinning the development of NR have not been extensively studied. Notably, most NR regress and only some develop into WT [7]. Identifying molecular changes underpinning the transformation of NR to WT may aid the understanding of WT pathogenesis and guide the development of targeted therapies. Therefore, we performed a systematic review of the literature in which molecular analysis was completed in NR or in a combination of NR and WT. This systematic review presents the current knowledge of somatic molecular changes in NR. 

## 2. Materials and Methods

### 2.1. Search Strategy and Eligibility Criteria 

This review was written according to the Preferred Reporting Items for Systematic Reviews and Meta-Analyses (PRISMA) statement [19,20] and the protocol was not registered. PubMed, EMBASE and Cochrane were systematically searched for all the available literature on molecular changes in NR in the English language, published from 1990 to 2022. The full search strategy is provided in Table S1. Articles were included if somatic molecular changes in NR were studied, including case reports in which specific analysis for NR was performed. Studies without molecular analysis in NR, non-English literature, animal studies or germline mutation analyses were excluded. Three authors (T.B., A.M., R.K.) assessed all articles independently based on the in- and exclusion criteria. After a comparison and the consensus of the authors on potentially relevant articles, the remaining publications were screened for eligibility criteria, based on their full text. Controversies were resolved by consensus. 

### 2.2. Quality Assessment 

The “Standards for Reporting Diagnostic Accuracy 2015” (STARD 2015) checklist [20] was used to assess the quality of the included studies individually and is provided in Supplementary Table S2a. The included case reports were assessed using the case report guidelines (CARE checklist 2013), the checklist is presented in Supplementary Table S2b [21]. The included studies were further assessed with the Oxford Centre for Evidence-Based Medicine Levels of Evidence Classification rubric [22], independently by the three authors (T.B., A.M., R.K.), for methodologic quality. In case of disagreement, it was solved by reaching consensus.

### 2.3. Data Extraction 

From the included articles, the number of NR, WT, WT-NR pairs analyzed, mean age at time of diagnosis of WT, sex, histological type of WT and NR and whether a WT was bilateral or hereditary, were obtained. Furthermore, from each study, the genes or chromosomal regions investigated with associated molecular techniques were extracted.

## 3. Results

### 3.1. Search Strategy and Eligibility Criteria 

**Figure 2:**
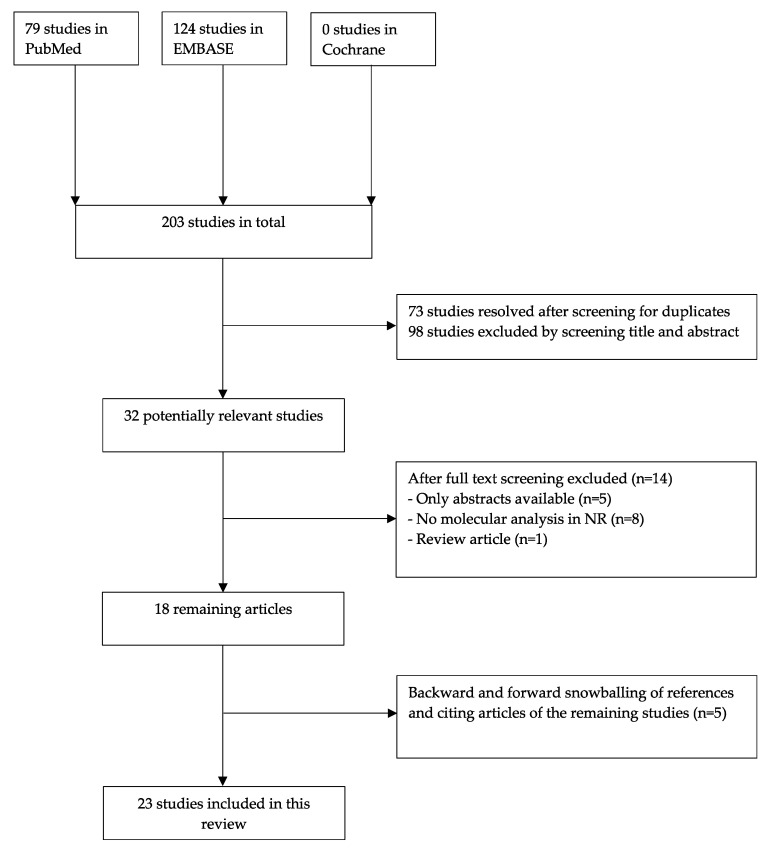
Flowchart of the search and selection process.

Our search in databases PubMed, EMBASE, and Cochrane generated a total of 203 articles. Seventy-three were duplicate articles that were found with more than one search engine and were therefore removed. The 130 remaining articles were screened for title and abstract after which 98 articles were excluded because of the in 2.1 mentioned criteria (Figure 2). We performed full-text screening in 32 potentially relevant studies, critically examining the in- and exclusion criteria. Consequently, 14 more articles were excluded, of which five were abstracts only and nine others were excluded since they did not meet the inclusion criteria. The remaining 18 articles were included in this systematic review. There were five additional articles identified by forward and backward snowballing, resulting in a total of 23 articles. In Figure 2, a flowchart of the search and selection process is presented based on the PRISMA scheme.

### 3.2. Quality Assessment 

Quality assessment was completed for all 23 included articles. An overview of the quality assessment of 18 included articles, using the STARD checklist, is provided in Supplementary Table S3a (including the STARD Checklist in Table S2a). The remaining five case reports, using the CARE checklist, are presented in Supplementary Table S3b. All included articles could be classified as Oxford level 2 to 4, as shown in Table 1. 

### 3.3. Characteristics of the Included Studies 

Patient characteristics of the 23 included studies are presented in Table 1. In those studies, 221 NR were analyzed, of which 53 were ILNR, 136 were PLNR, 11 were nephroblastomatosis and 21 were not further specified. A total of 119 WT-NR pairs were analyzed with different methods including methylation analysis, sequencing analysis, LOH analysis, expression analysis and in situ hybridization. There were 28 WT-NR pairs reported with concomitant bilateral WT, whereas this was not explicitly reported in the other cases. Sex and age were described in only a few studies. In the eight studies describing sexual gender, there were 42 females and 30 males. The age at diagnosis for WT was variable ranging from 11 months to 144 months and for NR from 10 months to 192 months. 

### 3.4. Chromosomal Changes 

Table 2 presents the somatic changes that were found in NR, including the gene or chromosomal region that was involved and the detection method that was used. We identified seven studies in which LOH analysis was used. LOH of 11p15 was found in 13/64 investigated NR (20%), and the same loss was confirmed in the corresponding WT (n = 13) [31,38]. Three studies demonstrated LOH of 11p13, which revealed that this was present in 4/28 WT-NR pairs (14%) all representing ILNR. There was a single WT-PLNR pair that showed LOH of 11p13 in the WT only [35,38,40]. Hoban et al. described a case in which the LOH of chromosome 11 (both 11p13 and 11p15) was observed, where in fact all other maternal chromosomal loci were lost in the NR and WT in a Beckwith–Wiedemann patient [42]. Three studies involved LOH of 16q showing loss in 13/53 WTs (25%). In two studies, loss of 16q was not found in their associated NR [32,38], but Austruy et al. detected loss of 16q in one case of nephroblastomatosis [41]. LOH of 7p was investigated in two pairs of NR-WT, but LOH of 7p was only present in WT, not in the corresponding NR [32,37].

Furthermore, genome-wide comparative genome hybridization (CGH) was performed in two other studies. Vuononvirta et al. investigated 50 PLNR and divided them into three subgroups. The first group showed no copy number changes at all (44%). The second group contained eight cases in which whole chromosome changes were observed (16%). The remaining group, involving 20 cases, presented multiple partial chromosomal gains or losses (40%). In 76%, the NR contained part of the copy number changes seen in the corresponding WT [31]. Steenman et al. also performed whole genome analysis by CGH. This revealed losses of 1p, 7p, 4q and gains of 1q and 12q in nephroblastomatosis and their associated WT. Loss of 11p was found in nephroblastomatosis only. Changes only present in WT were loss of 9p and gain of 8, 10q and 18. Loss of 16q was detected in one case of WT and in one case of nephroblastomatosis adjacent to WT [40].

MdZin et al. found loss of chromosome 22 and showed that the frequency of chromosome 22 loss depended on NR morphology. Dormant, involuted and sclerosing PLNR presented monosomy 22 in 30%, whereas the hyperplastic and adenomatous PLNR showed monosomy 22 in 50%, increasing to a rate of 60–80% in WT [28]. 

### 3.5. Structural Aberrations in Candidate Gene Studies 

While LOH analysis allows for detecting larger chromosomal regions without exactly identifying the responsible gene(s), candidate gene analysis by targeted sequencing might highlight abnormalities in putative oncogenes and tumor suppressor genes. Seven studies investigated single nucleotide changes in NR compared to WT. Four studies found mutations in the *WT1* gene and showed that these can be present in both NR and WT [35,38,43,45]. Using Sanger sequencing analysis, Fukuzawa et al. showed that *CTNNB1* mutations occurred only in WT (n = 6) and not in paired NR (n = 6) [35]. Another gene that may play a role in WT tumorigenesis, *WTX*, presented mutations in both NR (n = 1) and WT (n = 4) [29]. A *KRAS* gene mutation was found in one case of WT and adjacent NR in the setting of mosaicism [23,24]. In this same patient Slack et. al. [24] also described a somatic *FBXW7* mutation in two WT but not in another WT nodule or the associated NR.

Two studies reported an absence of mutations of specific candidate genes. In the first study by Wegert et al., *EGFR*-internal tandem duplications (ITD) and *BRAF*-internal deletions (ID) were investigated, involving 208 WTs and 12 NR, but no changes in any of these genes were found [26]. In the second study, where *PTEN* was investigated, no abnormalities were found in the WT and NR included in the study by Grill et al. [30].

### 3.6. Epigenetic Studies

Epigenetic changes were described in ten studies, and six of them focused on DNA methylation changes. The different platforms used for methylation profiling in these studies included bisulfate sequencing [34], combined bisulfate and restriction analysis (COBRA) [32,33], pyrosequencing [31], and Illumina BeadChip microarray [25,27]. Charlton et al. [27], using the latter technique, performed a longitudinal study to find differences in DNA methylation to compare NR to NK as well as to WT. For this purpose, differentially methylated regions (DMR) were compared and shown to differ between WT, NR and NK [46]. Hypermethylation was seen in 55% of 629 differentially methylated regions (DMR) in NR as compared to NK [27]. When NR were compared to WT, in paired analysis, two subgroups of WT could be distinguished. In one group, NR and WT grossly harbored the same epigenetic profiles. Yet, the other group of WT showed hypervariability in the methylation profiles in comparison to NR, suggesting that there is a shift in methylation during the development from NK to NR and/or WT [27]. 

Vuononvirta et al. [31] used pyrosequencing to analyze *H19*. Hypermethylation of this gene is associated with LOI of *IGF-2*, which was found in 23/33 (70%) cases and hypermethylation of *H19* in 37/40 (93%) of the PLNR cases. Brown et al. analyzed *H19* in relation to LOI at 11p13 and 11p15. They found *H19* hypermethylation in two WT-NR pairs using COBRA [32]. LOI at 11p13 and 11p15 was found in both WT-NR pairs. LOI of 11p13 leads to the decreased methylation of *WT1* antisense regulatory region (ARR), which results in LOI of the noncoding antisense RNA *WT1*-AS and the alternate coding *WT1* transcript A*WT1*. LOI of 11p15 is responsible for *IGF-2* overexpression, another imprinted gene on chromosome 11 [32]. Hypomethylation of *WT1* ARR in WT was also studied by Hancock et al. in two WT-NR pairs using bisulfate sequencing, revealing the lowest methylation levels in WT, the highest in NK, and NR showing methylation percentages between fetal kidney (FK) and NK. They also looked at the expression of A*WT1* and *WT1*-AS, and found biallelic expression of A*WT1* and *WT1*-AS in NR and WT, where monoallelic expression was found in the NK [34].

Cui et al. studied RNA expression of the *H19* gene in association with *IGF-2* expression, using in situ hybridization (ISH) in WT, NR and associated renal medulla. H19 was not expressed in WT and NR, but was present in normal renal medulla [39]. Yun et al. also investigated IGF-2 by in situ hybridization and Northern blotting in NR and WT. Both studies displayed comparable patterns in NR and WT, but there was a variable and heterogeneous level of expression. *IGF-2* expression was frequently associated with blastema [39,44]. LOI of *IGF-2* was studied by Ravenel et al. and was present in a WT–NR pair with two PLNR [36]. 

Coorens et al. used Illumina BeadChip microarray to demonstrate the presence of *H19* hypermethylation in NK with clonal expansions (58%), while this hypermethylation was not found in NK without clones. One WT–NR pair was included, in which the WT and NR emerged from a similar ancestral clone at different time points, which is indicative of an association between clones, NR and WT all showing *H19* hypermethylation [25]. 

Finally, Chilukamarri et al. investigated the *GLIPR1/RTVP-1* gene. Hypomethylation of this gene was shown in 21 out of 24 WTs. There were two associated NR analyzed, which also showed hypomethylation of this gene [33].

## 4. Discussion

We have presented an overview of all molecular studies on nephrogenic rests between 1990 and 2022. As a result of this long-time frame, a wide range of techniques were used to examine chromosomal regions, copy number variations, individual genes and epigenetic changes in NR. A total of 23 studies were found showing loss of chromosomal arms 11p13 and 11p15, 1p, 4q and 11p, and gains in 1q, 7q and 12q, as well as mutations in *WT1*, *WTX* and *KRAS* to occur in both NR and WT, suggesting these are early events (Table 3). Mutations in *CTNNB1* and *FBXW7* and LOH of 16q and 7p are only present in WT, but not the associated NR, therefore likely representing late events (Table 3). Furthermore, differential methylation levels display a relationship between WT, NR, and NK.

Summary of early molecular events that occur in both NR and WT and late molecular events that occur in WT only. Late events are therefore likely not involved in the progression from NR to WT. 

**Figure 3:**
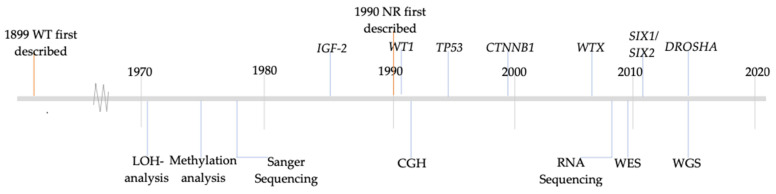
History of identified aberrations in WT and introduction of molecular methods. The above timeline shows selected WT-associated genes and there first description in relation to WT. The lower part indicates the year of the first description of the selected molecular methods. *LOH*, loss of heterozygosity; *CGH*, comparative genomic hybridization; *RNA*, ribonucleic acid; *WES*, whole exome sequencing; *WGS*, whole genome sequencing.

Little is known about the molecular pathogenesis of WT, including the mechanisms that affect transition from NR to WT, although NR have been recognized as precursor lesions [3]. Studying molecular changes in pairs of NR and WT might shed light on the timing of such events. If the same alteration occurs in NR and the associated WT, this can be considered as an early event. Indeed, LOI and LOH at 11p13 and 11p15 were found in both NR and WT, representing the chromosomal regions where the *WT1* and the *IGF2* genes are located, respectively [31,32,35,38,40]. No such gene correlations are known for the other chromosomal arms that were recurrently lost (1p, 4q, 7p and 11p), or gained (1q, 7q and 12q) [40]. MdZin et al. found an increasing frequency of monosomy of chromosome 22 from sclerotic/dormant PLNR to hyperplastic/adenomatous PLNR and then finally toward WT, suggesting that loss of chromosome 22 is an early event and that tumor suppressor genes on this chromosome might be involved in WT tumorigenesis [28]. Mutations in *WT1* [35,38,43,45] and *WTX* [29] can also be considered early events, as they were found in both NR and WT. *WT1* was first investigated in WT in 1991 [45,47], and *WTX* was first described in association with WT in 2007 (Figure 3) [48].

Genetic changes that are found in WT only, and not in NR, are likely to be late events and therefore not involved in the transition from NR to WT. For instance, LOH of 16q appears to be a late event [32,38,40,41]. However, in one case, loss of 16q was also found in the associated nephroblastomatosis [41]. Loss of 7p appears to occur later in development as well [32,37], although Steenman et al. found LOH of 7p in a case of nephroblastomatosis [40]. Furthermore, *CTNNB1* mutations, which were first described in WT in 1999 (Figure 3), are considered late events in Wilms tumorigenesis [29,35]. No *PTEN*, *EGFR* and *BRAF* mutations or rearrangements were found in both WT and NR, implying that these genes do not seem to contribute to the transition of NR to WT [26,30].

It should be noted that, due to the long time interval, there was large variation between studies with regard to the number and type of polymorphic markers used for LOH or LOI analyses, which may have had an effect on the detection of molecular abnormalities. Likewise, for the candidate gene studies, not all genes have been investigated completely or information on the extent of screening is lacking. In one study, all exons of *CTNNB1* were sequenced [35], while Park et al. [43] examined exons 2–10 for detecting mutations in *WT1*. In studies describing *WT1*, two studies used primers for only two exons to detect mutations in *WT1*, not mentioning if all other exons of *WT1* were sequenced [35,38]. In one other study that examined both *CTNNB1* and *WTX*, it was not reported which exons were sequenced [29]. Furthermore, many of the genes that have been shown to be involved in WT tumorigenesis, such as *SIX1*, *SIX2*, and *DROSHA*, have not been systematically analyzed in NR [49]. Thus, comprehensive analyses should be performed using WES or WGS to examine all genes and to prevent important genes from not being detected. 

Regarding epigenetic changes, there appears to be a correlation between the methylation and expression patterns of NR as compared to WT. The similar or increased methylation of WT and NR with respect to NK suggests that this might play a role in tumorigenesis [27]. There was increased methylation of *H19* in WT and NR, and also in the clonal expansions of NK, which suggests a transformation from NK to WT in the levels of methylation of *H19*. It is remarkable that the WT–NR pair included in this study arose from the same clone and that they are therefore phylogenetically related [25]. Together this may indicate that the clonal beds of NK are “primed” to become a WT. LOI at 11p15 and 11p13 and LOI of *IGF-2* were detected in WT and NR, and thus seem to be early events [32,36]. The presence of LOI of 11p13 correlates with the decreased methylation of *WT1* ARR, suggesting that imprinting defects at 11p13 may be involved in tumorigenesis [32,34]. *H19* and *IGF-2* are two parentally imprinted genes on 11p15, with opposite regulatory mechanisms: if *H19* is silenced, *IGF-2* on the other hand is upregulated [50,51]. This explains why there was expression of *IGF-2* in both NR and WT, while *H19* expression was not detected [39]. Together with ISH studies showing variable *IGF-2* expression patterns in NR and WT, these data suggest a role for *IGF-2* as an early driver of WT development [39,44]. Likewise, hypomethylation of *GLIPR1*/*RTVP1* was found in WT and NR, suggesting that this specific change might also contribute to WT development [33].

As previously described, NR are present in approximately 40% of unilateral WT and in almost all cases of bilateral WTs [14]. Nephroblastomatosis is more frequently present in bilateral WT [52]. This suggests that not every WT might arise from NR. Conversely, not every NR develops into a WT either. WT and NR both develop from the same embryonic tissue, and both morphologically reflect embryonal renal tissue. Normally, nephrogenesis stops at 34 weeks of gestation and any remaining nephrogenic tissue is considered NR. However, little is known about the underlying factors that lead to such persistence. ILNR are frequent in tumors with stromal histology that are typically associated with aberrations in *WT1* on chromosome locus 11p13. PLNR occur more often in blastemal and/or epithelial type WT and are associated with alterations in *IGF-2* on 11p15 [18]. These associations have been confirmed in several included studies where a distinction was made between PLNR and ILNR. *WT1* mutations and LOH of 11p13 are found in ILNR [35,38,43], and LOI of *IGF-2* in PLNR [36]. However, LOH of 11p15 can also be found in ILNR [38]. Interestingly, there is a known association between *WT1* and *CTNNB1* mutations in WT [53,54]. However, it is notable that *CTNNB1* mutations occur as late events and are only present in WT [35], while *WT1* mutations can occur in NR and WT [35,38,43,45]. Even though NR are precursors of WT, there are only few studies which focus on and describe the molecular pathogenesis of NR. In this review, all studies between 1990 and 2020 which analyzed molecular changes in NR and used molecular techniques were included, resulting in only 23 relevant articles, i.e., less than 1 per year over the investigated time frame. We chose 1990 as a starting point (Figure 3), as it coincides with the first histopathologic description of NR and with the wider applicability of molecular methods in biomedical research, but even until 2000 few molecular studies were performed. In addition, most techniques required frozen lesional tissue, while frequently only formalin-fixed paraffin embedded (FFPE) material was available, limiting the possibilities of molecular analyses, at least in previous decades. Finally, it is virtually impossible to distinguish NR macroscopically, apart from patients with nephroblastomatosis, requiring expert microscopic detection on FFPE material with its inherent limitations.

Throughout this review, we have applied the histological definition of NR and nephroblastomatosis over the radiological definition, which is not 100% superimposable with the histological term of NR. The supplied histological material was used as a reference point. The diagnosis of NR and WT can only be made after histological confirmation. On CT imaging, NR and nephroblastomatosis may show distinct features from WT, mainly with regard to the shape and size of the lesion [13]. However, up to 25% of the NR and WT are still misdiagnosed, with half of nodules radiologically diagnosed as WT, turning out be NR after histological confirmation [12]. It is outside the scope of this review to further discuss the potential a newer imaging technology, such as diffusion-weighted imaging (DWI) MRI for the above distinction.

As presented in Table 1, the number of WT and NR examined varied greatly from study to study. Some genes have been studied on less than five tumors. Therefore, firm conclusions cannot be drawn from these studies. To do so in the future, larger-scale studies of WT and corresponding NR would be needed to assign certain genes a significant role in the development of NR to WT. In addition, mutations such as *DROSHA* and *SIX1* and *SIX2*, which are known driver mutations in WT, have not been examined in NR. These genes, as can be seen in Figure 3, have been discovered from 2010 onwards. Many studies described in this review were completed at a time when these candidate genes were not yet identified and have therefore not been investigated in NR. 

Until 2000, mRNA in situ hybridization (ISH), sequencing analysis, LOH analysis and CGH analysis were used to analyze NR (Figure 3). From 2006 methylation studies emerged in which the difference in methylation patterns between NR and WT was investigated [25,27,31,32,33,34]. Several methods that were used in studies presented in this review may currently be considered outdated with the advent of new molecular methods, including whole exome and whole genome sequencing, RNA sequencing and also spatial transcriptomics, which allows tissue context while analyzing gene expression profiles. These techniques may allow faster progress and seem particularly relevant for NR analysis, which still relies on morphological identification. It is important to consider tests for the early detection of genetic, epigenetic and somatic changes in NR that may become clinically relevant to the patient before NR develop into a WT. At the moment, not all genetic predispositions have been explored. Therefore, all children with WT or nephroblastomatosis should be referred to a clinical geneticist and advised to undergo whole exome or whole genome sequencing, as there is a high risk (up to 33%) of genetic predisposition, as was recently shown in a Dutch cohort [55]. This certainly applies to children with bilateral WT, in whom an 80% chance of genetic predisposition has recently been revealed [56]. 

## 5. Conclusions

In conclusion, over the past 30 years, several studies have looked at NR with or without associated WT. As a result, much has become known about the genetic changes in NR. LOH of 11p13 and 11p15, expression of *IGF-2* and mutations in *WT1* and *WTX* appear to play a role in the early tumorigenesis of WT. LOH of 16q and 7p and mutations in *CTNNB1* seem to occur later in development. Methylation patterns of NR in comparison to WT appear to be similar. Due to rapid advances in (genome-wide) molecular techniques, the increased possibilities to use FFPE material and the availability of histologically confirmed frozen material, genetic changes in NR and corresponding WT may be investigated in larger series and might unravel early steps in the progression of WT tumorigenesis.

## Figures and Tables

**Table 1:** Characteristics of reviewed NR cases.

Study	Oxford Level	NR (n)	NR Type	WT (n)	WT Type	WT-NR Pairs	Bilateral WT	Sex	Age at Diagnosis
Chang et al., 2021 * [23]	4	Multiple 1	PLNR NBL	1 -	epithelial type -	1 -	Yes -	M F	21 months 36 months
Slack et al., 2021 [24]	4	Multiple	PLNR	1	epithelial type	1	Yes	M	21 months
Coorens et al., 2019 [25]	4	1	ND	23	ND	1	No	ND	ND
Wegert et al., 2018 [26]	2	12	ND	208	ND	ND	ND	ND	ND
Charlton et al., 2015 [27]	2	22	17 PLNR, 5 ILNR	36	ND	20	ND	ND	ND
MdZin et al., 2011 [28]	4	9 1	9 PLNR 1 PLNR	2 1	blastemal type mixed type	1 1	Yes No	M M	42 months 48 months
Fukuzawa et al., 2010 [29]	4	4	4 ILNR	4	3 stromal type, 1 blastemal type	4	ND	3F, 1M	ND
Grill et al., 2010 [30]	2	26	18 PLNR, 8 ILNR	22	14 mixed type, 2 stromal type, 4 blastemal type and 1 regressive type, 1 not known	22	ND	ND	ND
Vuononvirta et al., 2008 [31]	2	50	50 PLNR	25	ND	25	17/50 NR	ND	ND
Brown et al., 2008 ** [32]	4	Multiple	PLNR	51	ND	2	2; 2 WT-NR pairs	ND	ND
Chilukamarri et al., 2007 [33]	4	2	ND	24	5 stromal type, 1 epithelial type, 4 blastemal type, 8 mixed type, 6 not known	2	5; 2 WT-NR pairs	8M, 16F (WT-NR pairs 2F)	WT 36 ^ months
Hancock et al., 2007 [34]	2	Multiple	ND	2	mixed type	2	2 WT-NR pairs	ND	ND
Fukuzawa et al., 2006 [35]	4	3	3 ILNR	2	stromal type	2	ND	ND	ND
Ravenel et al., 2001 [36]	4	2	2 PLNR	60	ND	1	ND	ND	ND
Powlesland et al., 1999 [37]	2	2	PLNR	7	3 mixed type, 1 stromal type, 3 blastemal type	2	2, 1 WT-NR pair	4 M, 3F (WT-NR pair M)	WT 43 months ^
Charles et al., 1998 [38]	2	42	22 PLNR, 17 ILNR, 3 both types	139	ND	ND	9; 2 WT-NR pairs	ND	NR 49 months ^ PLNR 54 ^, ILNR 44 ^
Cui et al., 1997 [39]	2	8	7 PLNR, 1 ILNR	14	ND	7	ND	ND	ND
Steenman et al., 1997 [40]	2	7	7 NBL	46	ND	6	2, no WT-NR pairs	ND	ND
Austruy et al., 1995 [41]	2	2	NBL	28	ND	2	3, no WT-NR pairs	ND	ND
Hoban et al., 1995 [42]	4	Multiple	PLNR, ILNR	1	ND	1	ND	F	11 months
Park et al., 1993 [43]	4	Multiple	1 ILNR, multiple PLNR	19	blastemal type	2	ND	2F (WT-NR pair)	11 months and 48–84/144 months
Yun et al., 1993 [44]	2	15	13 ILNR, 2 PLNR	31	6 blastemal type, 5 stromal dominant, 20 mixed type	15	ND	14M, 17F (WT-NR pairs 8M, 7F)	WT 43 months ^
Pritchard-Jones et al., 1991 [45]	2	Multiple	NBL and not known	32	18 mixed type, 4 epithelial type and 10 not known	ND	ND	ND	ND

NR, nephrogenic rests; WT, Wilms tumors; n, population size expressed as number of NR or WT; ND, no (complete/clear) data; PLNR, perilobar nephrogenic rests; ILNR, intralobular nephrogenic rests; NBL, nephroblastomatosis; ^, mean; *, one case already reported by Slack et al.; **, two cases already reported by Hancock et al.

**Table 2:** Genetic changes in NR.

Study	Gene/Chromosomal Region	Method	Results	Conclusion
Chang et al., 2021 [23]	*KRAS*, *FBXW7*	NGS/WGS	Association between nephroblastomatosis and *KRAS.*	Possible association between *KRAS* and bilateral WT and between mosaic *KRAS* and NBL.
Slack et al., 2021 [24]	*KRAS*, *FBXW7*	NGS	Mosaic *KRAS* has similar frequencies in WT and adjacent NR. Bilateral WT, but not two adjacent NR, contained the *FBXW7* mutation.	Similar *KRAS* allele frequencies in WT and NR. *FBXW7* mutation seems to be a late event in WT tumorigenesis
Coorens et al., 2019 [25]	Genome wide	WGS, WES Methylation analysis	Clonal nephrogenesis in 14/23 (61%) WTs (4/4 bilateral). WT and NR from same patient arose at different times from the same ancestral clone. *H19* hypermethylation in 7/12 NK with clonal nephrogenesis but not in NK without clonal nephrogenesis.	There is an association between VAF of embryonal clonal expansions, *H19* hypermethylation and development of WT.
Wegert et al., 2018 [26]	*EGFR*, *BRAF*	WGS	No mutations in both NR (n = 12) and WT (n = 208)	No *EGFR, BRAF* mutations in NR and WT
Charlton et al., 2015 [27]	Genome wide	Comprehensive methylome analysis	NR vs. NK: 629 DMR, 55% showed hypermethylation. NR vs. WT: 2 subgroups WT, one group showed the same epigenetics as NR and one group presented increased methylation variability.	Methylation profiles vary significantly between NK, NRs and WTs and alterations in the methylome lead to NR formation and transformation to WTs.
MdZin et al., 2011 [28]	Chromosome 22	FISH and microsatellite analysis	Dormant, involuted and sclerosing NR displayed monosomy 22 in 30%, hyperplastic and adenomatous NR in 50%, and 60–80% in nuclei of WT.	More common loss of chromosome 22 in the development of PLNR (from dormant to hyperplastic) to WT.
Fukuzawa et al., 2010 [29]	*WTX*, *CTNNB1*	Sequencing analysis, MLPA/microsatellite analysis	*CTNNB1* mutation: n = 0 in NR, n = 4 in WT. *WTX* mutation: n = 1 in NR, n = 4 in WT.	*WTX* can occur as an early event, or in later stages of development, *CTNNB1* is a late event.
Grill et al., 2010 [30]	*PTEN*	LOH-analysis, sequencing analysis	None of the WT and none of the ILNR/PLNR showed LOH.	*PTEN* does not play a role in tumorigenesis. Downregulation does not cause WNT-pathway activation.
Vuononvirta et al., 2008 [31]	Genome wide	aCGH LOH-analysis Methylation analysis	PLNR 3 groups: no copy number changes (44%); single, whole chromosome changes (16%); multiple gains or losses (40%). In 76% NR changes correspond to WT. 11p15 LOH in 10/39 (26%), in NR and tumor (n = 9). H19 hypermethylation in 37/40 (93%) PLNR.	PLNR are non-obligate precursors of WT.
Brown et al., 2008 [32]	LOI 11p13/15, LOH 16q, 7p	LOH-analysis Methylation analysis	LOI 11p13 and 11p15; LOH 7p/16q not in NR, but in WT. H19 DMR analysis increased in NR and WT (n = 2). Reduced methylation of *WT1* ARR in NR and WT.	LOI at 11p13 and 11p15 are early events in NR, and occur prior to LOH at 7p or 16q. *H19* DMR methylation was increased in NR and WT.
Chilukamarri et al., 2007 [33]	*GLIPR1/RTVP-1*	Methylation analysis	Hypomethylation WT (21/24) and NR (n = 2)	Hypomethylation of *GLIPR1*/*RTVP1* may play a role in WT tumorigenesis.
Hancock et al., 2007 [34]	*WT1*	Methylation and expression analysis	WT showed hypomethylation of WT1 ARR on both alleles. WT1 methylation differs between FK, NR and WT.	Imprinting defects at 11p13 contribute to WT tumorigenesis.
Fukuzawa et al., 2006 [35]	*CTNNB1*, *WT1* 11p13	Sequencing analysis LOH-analysis	No *CTNNB1* mutations in NR, n = 2 in WT. *WT1* mutation: n = 3 in NR and n = 2 in associated WT. 11p13 LOH in ILNR and tumor (n = 1)	Mutations in the *CTNNB1* occur in the later stages of WT tumorigenesis. Mutations of *WT1* are early events in ILNR.
Ravenel et al., 2001 [36]	*IGF-2*	Expression analysis	LOI of *IGF-2* in 2 PLNR and associated WT.	LOI of *IGF-2* seems to be an early event in the development of WT.
Powlesland et al., 1999 [37]	7p	LOH-analysis	LOH of 7p in 7/77 WT, one associated NR has no LOH of 7p.	LOH of 7p seems to be a late event in WT tumorigenesis.
Charles et al., 1998 [38]	*WT1* 11p15, 11p13, 16q	Sequencing analysis LOH-analysis	Two pairs of ILNR and WT showed *WT1* mutation. LOH at 11p15 in 3/25 (12%, all ILNR). LOH at 11p13 in 3/26 (12%, 2 in ILNR, in case with PLNR only in tumor). Loss of 16q in 4/23, only in tumors.	LOH at 11p13 and 11p15 is seen in ILNR and WT. PLNR showed no LOH 11p events occur early in WT development. Genetic changes at 16q are a late event.
Cui et al., 1997 [39]	*H19*, *IGF-2*	ISH	*IGF-2* expression present in NR, WT and kidney medulla. *H19* is not expressed in NR and WT in contrast to renal medulla. Pattern of *IGF-2* expression differs in NR and WT.	Association between expression of *IGF-2* and *H19*, but *H19* inactivation also without effect on *IGF-2* expression status. Loss of *H19* expression possibly involved in blastemal overgrowth.
Steenman et al., 1997 [40]	Genome wide	CGH	Losses in 1p, 4q, 7p and gains in 7q, 1q and 12q can occur in both tumor and NBL, even as LOH at 1p and 11p13; loss of 11 only in NBL; loss of 9p, 16q and gain of 8, 10q and 18 are only seen in WT.	Two specific 1p regions involved in WT etiology.
Austruy et al., 1995 [41]	16q	LOH analysis	LOH of 16q in 7/28 WT and in one out of two associated NBL.	LOH of 16q can also occur as an early event Wilms tumorigenesis.
Hoban et al., 1995 [42]	Genome wide	LOCH analysis	LOCH present on chromosome 11 (11p13/p15), but also on all other chromosomes.	Loss of all maternal loci, including #11, suggests to be an early genetic event.
Park et al., 1993 [43]	*WT1*	Sequencing analysis	*WT1* mutations in both cases in NR and WT (n = 2), both somatic.	*WT1* inactivation seems to be an early genetic event.
Yun et al., 1993 [44]	*IGF-2*	mRNA ISH	*IGF-2* hybridization patterns of NR equal to WTs. *IGF-2* expression in NR variable. *IGF-2* transcripts more frequent in tumors with blastema.	Occasional NR also displayed different *IGF-2* expression, suggesting NR could be precursor lesions of WT.
Pritchard-Jones et al., 1991 [45]	*WT1*	mRNA ISH	NBL have high levels of expression, similar to WT.	*WT1* contributes to WT tumorigenesis.

NGS, next generation sequencing; WGS, whole genome sequencing; WES, whole exome sequencing; NK, normal kidney; VAF, variable allele frequency; DMR, differently methylated regions; FISH, fluorescent in situ hybridization; MLPA, multiplex ligation dependent probe amplification; LOH, loss of heterozygosity; aCGH, array comparative genomic hybridization; LOI, loss of imprinting; *WT1* ARR, *WT1* antisense regulatory region; FK, foetal kidney; ISH, in situ hybridization. CGH, comparative genomic hybridization; LOCH, loss of constitutional heterozygosity.

**Table 3:** Early and late events.

Early Events	Late Events
LOI and LOH of 11p13 LOI and LOH of 11p15 Loss of 1p, 4q and, 11p Gains in 1q, 7q and 12q Loss of chromosome 22 Mutations in *WT1*, *WTX* and *KRAS*	LOH of 16q LOH of 7p Mutations in *CTNNB1* and *FBXW7*

## Data Availability

No new data were created in this study. Data sharing is not applicable to this article.

## Supplementary Materials

The following supporting information can be downloaded at: https://www.mdpi.com/article/10.3390/cancers15051363/s1, Table S1: Search strategy PubMed and EMBASE, Table S2a: “Standards for Reporting Diagnostic Accuracy 2015” (STARD 2015) checklist, Table S2b: Case report guidelines (CARE checklist 2013), Table S3a: Quality assessment of 18 out of 23 included articles based on the STARD 2015 checklist, Table S3b: Quality assessment of 5 out of 23 included articles based on the CARE 2013 checklist.
