# Supplementary material for: Somatic, Genetic and Epigenetic Changes in Nephrogenic Rests and Their Role in the Transformation to Wilms Tumors, a Systematic Review
Source: Cancers (Basel). 2023 Feb 21;15(5):1363. doi: 10.3390/cancers15051363 (PMC10000075; doi:10.3390/cancers15051363)
Supplement: Supplementary file 1 [file cancers-15-01363-s001.zip › cancers-2117571-supplementary.pdf]

Supplementary Table S1. Search strategy PubMed and EMBASE

PubMed full search

(“nephroblastomatosis”[Title/Abstract] OR “nephrogenic rest*”[Title/Abstract])
AND
(“molecular biology”[MeSH Terms] OR “molecular” [Title/Abstract] OR “biochemical”[Title/Abstract] OR “biomarker*”[Title/Abstract] OR “mutation*”[Title/Abstract])

Last performed: 25th of January 2022
Total of 79 results

EMBASE full search

'nephroblastomatosis'/exp OR 'nephroblastomatosis' OR 'nephroblastomatosis':ti,ab,kw OR 'nephrogenic rest*':ti,ab,kw
AND
'biochemistry'/exp OR 'biochemistry':ti,ab,kw OR 'mutation'/exp OR 'mutation':ti,ab,kw OR 'molecular biology'/exp OR 'molecular biology':ti,ab,kw OR 'molecular':ti,ab,kw OR 'biomarker'/exp OR 'biomarker':ti,ab,kw

Last performed: 25th of January 2022
Total of 124 results (without MEDLINE)

Supplementary Table S2a. “Standards for Reporting Diagnostic Accuracy 2015” (STARD 2015) checklist

Section & Topic	No	Item	Reported on page #
**TITLE OR ABSTRACT**			
	**1**	Identification as a study of diagnostic accuracy using at least one measure of accuracy<br>(such as sensitivity, specificity, predictive values, or AUC)	
**ABSTRACT**			
	**2**	Structured summary of study design, methods, results, and conclusions<br>(for specific guidance, see STARD for Abstracts)	
**INTRODUCTION**			
	**3**	Scientific and clinical background, including the intended use and clinical role of the index test	
	**4**	Study objectives and hypotheses	
**METHODS**			
Study design	**5**	Whether data collection was planned before the index test and reference standard<br>were performed (prospective study) or after (retrospective study)	
Participants	**6**	Eligibility criteria	
	**7**	On what basis potentially eligible participants were identified<br>(such as symptoms, results from previous tests, inclusion in registry)	
	**8**	Where and when potentially eligible participants were identified (setting, location and dates)	
	**9**	Whether participants formed a consecutive, random or convenience series	
Test methods	**10a**	Index test, in sufficient detail to allow replication	
	**10b**	Reference standard, in sufficient detail to allow replication	
	**11**	Rationale for choosing the reference standard (if alternatives exist)	
	**12a**	Definition of and rationale for test positivity cut-offs or result categories<br>of the index test, distinguishing pre-specified from exploratory	
	**12b**	Definition of and rationale for test positivity cut-offs or result categories<br>of the reference standard, distinguishing pre-specified from exploratory	
	**13a**	Whether clinical information and reference standard results were available<br>to the performers/readers of the index test	
	**13b**	Whether clinical information and index test results were available<br>to the assessors of the reference standard	
Analysis	**14**	Methods for estimating or comparing measures of diagnostic accuracy	
	**15**	How indeterminate index test or reference standard results were handled	
	**16**	How missing data on the index test and reference standard were handled	
	**17**	Any analyses of variability in diagnostic accuracy, distinguishing pre-specified from exploratory	
	**18**	Intended sample size and how it was determined	
**RESULTS**			
Participants	**19**	Flow of participants, using a diagram	
	**20**	Baseline demographic and clinical characteristics of participants	
	**21a**	Distribution of severity of disease in those with the target condition	
	**21b**	Distribution of alternative diagnoses in those without the target condition	

	**22**	Time interval and any clinical interventions between index test and reference standard	
Test results	**23**	Cross tabulation of the index test results (or their distribution) by the results of the reference standard	
	**24**	Estimates of diagnostic accuracy and their precision (such as 95% confidence intervals)	
	**25**	Any adverse events from performing the index test or the reference standard	
**DISCUSSION**			
	**26**	Study limitations, including sources of potential bias, statistical uncertainty, and generalisability	
	**27**	Implications for practice, including the intended use and clinical role of the index test	
**OTHER INFORMATION**			
	**28**	Registration number and name of registry	
	**29**	Where the full study protocol can be accessed	
	**30**	Sources of funding and other support; role of funders	

Supplementary Table S2b. Case report guidelines (CARE checklist 2013)

CARE case report guidelines

CARE Checklist of information to include when writing a case report

(cc) BY-NC-ND

Topic	Item	Checklist item description	Reported on Line
Title	1	The diagnosis or intervention of primary focus followed by the words "case report"	
Key Words	2	2 to 5 key words that identify diagnoses or interventions in this case report, including "case report"	
Abstract (no references)	3a	Introduction: What is unique about this case and what does it add to the scientific literature?	
	3b	Main symptoms and/or important clinical findings	
	3c	The main diagnoses, therapeutic interventions, and outcomes	
	3d	Conclusion—What is the main "take-away" lesson(s) from this case?	
Introduction	4	One or two paragraphs summarizing why this case is unique (**may include references**)	
Patient Information	5a	De-identified patient specific information.	
	5b	Primary concerns and symptoms of the patient.	
	5c	Medical, family, and psycho-social history including relevant genetic information	
	5d	Relevant past interventions with outcomes	
Clinical Findings	6	Describe significant physical examination (PE) and important clinical findings.	
Timeline	7	Historical and current information from this episode of care organized as a timeline	
Diagnostic Assessment	8a	Diagnostic testing (such as PE, laboratory testing, imaging, surveys).	
	8b	Diagnostic challenges (such as access to testing, financial, or cultural)	
	8c	Diagnosis (including other diagnoses considered)	
	8d	Prognosis (such as staging in oncology) where applicable	
Therapeutic Intervention	9a	Types of therapeutic intervention (such as pharmacologic, surgical, preventive, self-care)	
	9b	Administration of therapeutic intervention (such as dosage, strength, duration)	
	9c	Changes in therapeutic intervention (with rationale)	
Follow-up and Outcomes	10a	Clinician and patient-assessed outcomes (if available)	
	10b	Important follow-up diagnostic and other test results	
	10c	Intervention adherence and tolerability (How was this assessed?)	
	10d	Adverse and unanticipated events	
Discussion	11a	A scientific discussion of the strengths AND limitations associated with this case report	
	11b	Discussion of the relevant medical literature **with references**.	
	11c	The scientific rationale for any conclusions (including assessment of possible causes)	
	11d	The primary "take-away" lessons of this case report (without references) in a one paragraph conclusion	
Patient Perspective	12	The patient should share their perspective in one to two paragraphs on the treatment(s) they received	
Informed Consent	13	Did the patient give informed consent? Please provide if requested	Yes ☐ No ☐

Supplementary Table S3a. Quality assessment of 18 out of 23 included articles based on the STARD 2015 checklist

STARD-item	1	2	3	4	5	6	7	8	9	10a	10b	11	12a	12b	13a	13b	14	15	16	17	18	19	20	21a	21b	22	23	24	25	26	27	28	29	30
Coorens et al. 2019	N	N	Y	Y	Y	Y	Y	U	U	Y	N	N	N	N	U	U	Y	N	N	N	N	N	N	N	N	N	N	Y	N	Y	Y	N	Y	Y
Wegert et al. 2018	N	N	Y	Y	Y	Y	Y	U	U	Y	Y	U	Y	N	U	U	Y	N	N	N	N	N	N	N	N	N	N	N	N	Y	Y	N	Y	Y
Charlton et al. 2015	N	Y	Y	Y	Y	Y	Y	U	U	Y	N	N	Y	N	U	U	Y	Y	Y	N	N	N	N	N	N	N	N	N	N	Y	Y	N	Y	Y
Grill et al. 2010	N	Y	Y	Y	Y	Y	Y	U	U	Y	N	N	N	N	U	U	N	N	N	N	N	N	N	N	N	N	N	N	N	Y	Y	N	N	Y
Fukuzawa et al. 2010	N	Y	Y	Y	Y	Y	Y	U	U	Y	N	N	N	N	U	U	N	N	N	N	N	N	Y	N	N	N	N	N	N	Y	Y	N	N	Y
Vuononvirta et al. 2008	N	Y	Y	Y	Y	Y	Y	U	U	Y	N	N	Y	N	U	U	Y	Y	Y	N	N	N	N	N	N	N	N	Y	N	Y	Y	N	N	Y
Brown et al. 2008	N	N	Y	Y	Y	Y	Y	U	U	Y	N	N	N	N	U	U	N	N	N	N	N	N	N	N	N	N	N	Y	N	Y	Y	N	N	Y
Chilukamarri et al. 2007	N	Y	Y	Y	Y	Y	Y	U	U	Y	N	N	N	N	U	U	Y	N	N	N	N	N	Y	N	N	N	N	N	N	Y	Y	N	N	Y
Hancock et al. 2007	N	Y	Y	Y	Y	Y	Y	U	U	Y	N	N	N	N	U	U	N	N	N	N	N	N	N	N	N	N	N	N	N	Y	Y	N	Y	Y
Fukuzawa et al. 2006	N	Y	Y	Y	Y	Y	Y	U	U	Y	N	N	N	N	U	U	Y	N	N	N	N	N	N	N	N	N	N	N	N	Y	Y	N	N	Y
Ravenel et al. 2001	N	Y	Y	Y	Y	Y	Y	U	U	Y	N	N	N	N	U	U	Y	N	N	N	N	N	N	N	N	N	N	Y	N	Y	Y	N	N	Y
Powlesland et al. 1999	N	Y	Y	Y	Y	Y	Y	U	U	Y	N	N	N	N	U	U	Y	Y	N	N	N	N	Y	N	N	N	N	N	N	Y	Y	N	N	Y
Charles et al. 1998	N	Y	Y	Y	Y	Y	Y	U	U	Y	N	N	N	N	U	U	Y	Y	N	N	N	N	Y	N	N	N	N	N	N	Y	Y	N	N	N
Cui et al. 1997	N	N	Y	Y	Y	Y	Y	U	U	Y	N	N	N	N	U	U	N	N	N	N	N	N	Y	N	N	N	N	N	N	Y	Y	N	N	Y
Steenman et al. 1997	N	Y	Y	Y	Y	N	N	U	U	Y	N	N	Y	N	U	U	N	N	N	N	N	N	Y	N	N	N	N	N	N	Y	Y	N	N	Y
Austruy et al. 1995	N	Y	Y	Y	Y	Y	N	U	U	Y	N	N	N	N	U	U	N	N	N	N	N	N	N	N	N	N	N	N	N	Y	Y	N	N	Y
Yun et al. 1993	N	Y	Y	Y	Y	N	N	U	U	Y	N	N	N	N	U	U	N	N	N	N	N	N	Y	N	N	N	N	N	N	Y	Y	N	N	Y
Pritchard-Jones et al. 1991	N	Y	Y	Y	Y	Y	N	U	U	Y	N	N	N	N	U	U	N	N	N	N	N	N	N	N	N	N	N	N	N	Y	Y	N	N	N

Y = Present, N = Absent, U = Unclear

Supplementary Table S3b. Quality assessment of 5 out of 23 included articles based on the CARE 2013 checklist

CARE-item	1	2	3a	3b	3c	3d	4	5a	5b	5c	5d	6	7	8a	8b	8c	8d	9a	9b	9c	10a	10b	10c	10d	11a	11b	11c	11d	12	13
Chang et al. 2021	N	N	Y	Y	Y	Y	Y	Y	Y	Y	N	Y	N	Y	N	Y	Y	N	N	N	N	Y	N	N	Y	Y	Y	Y	N	U
Slack et al. 2021	N	N	Y	Y	Y	Y	Y	Y	Y	Y	Y	Y	N	Y	N	Y	Y	N	N	N	N	Y	N	N	Y	Y	Y	Y	N	U
MdZin et al. 2011	N	N	Y	Y	Y	Y	Y	Y	Y	N	Y	N	N	Y	N	Y	Y	Y	N	Y	N	Y	N	N	Y	Y	Y	Y	N	U
Hoban et al. 1995	N	N	Y	Y	Y	N	N	Y	Y	N	N	N	N	Y	N	Y	Y	N	N	N	N	Y	N	N	Y	Y	Y	Y	N	U
Park et al. 1993	N	N	Y	Y	Y	N	N	Y	Y	Y	Y	N	N	Y	N	Y	Y	Y	N	N	N	N	N	N	Y	Y	Y	Y	N	U

Y = Present, N = Absent, U = Unclear
